# Association between DLA-DRB1.2 allelic diversity and development of mammary gland tumors in dogs

**DOI:** 10.1186/s13028-019-0491-z

**Published:** 2019-11-14

**Authors:** Seyed Milad Vahedi, Gholamreza Nikbakht, Shahram Jamshidi, Leila Lankarani, Nika Alimi, Atefeh Esmailnejad

**Affiliations:** 1 Department of Microbiology and Immunology, Faculty of Veterinary Medicine, University of Tehran, Tehran, Islamic Republic of Iran; 2 Department of Internal Medicine, Faculty of Veterinary Medicine, University of Tehran, Tehran, Islamic Republic of Iran; 3 Department of Pathobiology, School of Veterinary Medicine, Shiraz University, Shiraz, Islamic Republic of Iran

**Keywords:** Canine mammary gland tumors, MHC, DLA-DRB1.2, Dog

## Abstract

**Background:**

The major histocompatibility complex (MHC) is the best-characterized genetic region related to resistance/susceptibility to a wide range of infectious and immune-mediated diseases. Evidences suggest that MHC class II genes may play an important role in developing different types of tumors including breast cancer. Canine mammary gland tumors (CMTs) are the most common neoplasms in female dogs. In the current study, the association of canine MHC class II DLA-DRB1.2 genotypes with development of mammary gland tumor profiles in dogs was investigated. DLA-DRB1.2 allelic diversity was determined in 40 dogs (18 CMT cases and 22 controls) using HRM technique and DNA sequencing. Association of the DLA-DRB1.2 genotypes with CMT profiles was expressed as odds ratio (OR).

**Results:**

Based on the histopathological typing of tumors, CMT cases were categorized into 4 groups: simple carcinoma, complex carcinoma, carcinoma arising in a benign tumor and special types of carcinoma. A total of eight HRM profiles (A to H) were identified in dogs sampled. The association study revealed a significant correlation between DLA-DRB1.2 genotypes with different CMT profiles. The E genotype was significantly associated with increased risk of carcinoma arising in a benign tumor, and the B genotype represented a positive correlation with complex carcinoma. Significant association was also observed between the heterozygosity of DLA-DRB1.2 genotypes and decreased risk of developing tumor in dogs.

**Conclusions:**

These results provide additional support for the association between DLA-DRB1 genes and development of mammary gland tumors in dogs and could potentially be used for early diagnosis of neoplasia and identifying susceptible dogs.

## Background

Canine mammary gland tumors (CMTs) are considered as the most common neoplasm in female dogs. The majority of malignant mammary gland tumors in dogs are carcinomas, while sarcomas account for 5% [1]. Different factors are involved in development of mammary neoplasms including age, breed and genetic predisposition, hormones and growth factors, cyclooxygenase-2 expression, and diet [2]. Among these, the role of the genetic background in conferring the increased risk of developing mammary gland tumors is quite prominent [3].

The major histocompatibility complex (MHC) is a cluster of genes associated with immune responses to infectious diseases and immune-mediated disorders. MHC allele products bind to intracellular and extracellular antigens and present them to T cells to induce an effective immune response. The canine MHC, also known as dog leukocyte antigen (DLA), is composed of three clusters of genes, including class I, II and III genes [4]. DLA-class I and II molecules are responsible for presenting of self and non-self antigens to the immune system and regulating the immune responses [5]. DLA-class II region includes four loci DRA, DRB1, DQA1 and DQB1. Kennedy et al. [6] identified 90 DLA-DRB1, 22 DLA-DQA1 and 54 DLA-DQB1 alleles in the dog. The MHC class II alleles have been associated with a wide range of immune and non-immune responses in humans and animals and considered as a genetic risk factor for many autoimmune diseases [7]. It has been demonstrated that susceptibility and resistance to diseases, such as chronic superficial keratitis (CKS), hypoadrenocorticism and Greyhound meningoencephalitis are associated with the MHC class II alleles [8–10].

A strong association between MHC class II alleles and risk of developing different types of tumors has been reported in humans. Previous studies demonstrated the role of the HLA-DRB1 locus in genetic susceptibility to colorectal carcinoma, cervical cancer, acute leukemia, melanoma, renal cell carcinoma, and breast cancer [11–16]. Several other studies conducted in humans have also showed that some HLA-DRB1 genotypes may increase or decrease the risk of breast cancer [12, 17–19]. In spite of several studies on the association between DRB1 genotypes and breast cancer in human, to the best of our knowledge, there is no information regarding the MHC genotypes and CMTs. In this study, DLA-DRB1 genetic polymorphism was evaluated in a dog population. Subsequently, the association of MHC genotypes with mammary gland tumor profiles was analyzed.

## Methods

### Sample collection

This study was conducted prospectively on 18 bitches of six breeds with malignant CMTs referred to the Small Animal Teaching Hospital of University of Tehran, Iran, between 2012 and October 2017. A total of 22 breed and age-matched healthy bitches was considered as control group (Table 1). Tumors classification was carried out according to the World Health Organization’s criteria for histologic typing of CMTs [20]. The examined CMTs consisted of 8 (44.5%) complex carcinoma, 4 (22.2%) simple carcinoma, 4 (22.2%) carcinoma arising in a benign tumor and 2 (11.1%) special types of carcinoma. Blood samples from control dogs were collected in microtubes containing EDTA under sterile conditions and stored at − 20 °C until further analysis. The study was approved by Institutional Ethical Committee of University of Tehran, Tehran, Iran.

**Table 1:** Demographic characteristics of canine mammary tumor (CMT) cases and healthy control groups

	CMT (n = 18)	Controls (n = 22)	P-value
Age (years)	7.50	7.50	–
Breed			
Terrier	9 (50.0%)	10 (45.5%)	0.512
Shih Tzu	4 (22.2%)	5 (22.7%)	0.503
German Shepherd	2 (11.1%)	2 (9.1%)	0.617
Great Dane	1 (5.6%)	1 (4.5%)	0.450
Pekingese	1 (5.6%)	3 (13.6%)	0.558
Chihuahua	1 (5.6%)	1 (4.5%)	0.450

### Genomic DNA extraction

DNA extraction from dogs of the tumor group was achieved using paraffin embedded blocks. Sections of paraffin embedded blocks were deparaffinized in xylene, 100% ethanol, 96% ethanol and 70% ethanol. DNA extraction of deparaffinized tissue was performed according to the instruction of extraction kit (Tissue DNA extraction kit, MBST, Iran). Genomic DNA from control group blood samples was also extracted using AccuPrep Genomic DNA extraction kit (i-genomic Blood DNA Extraction Mini Kit, Intron, Republic of Korea).

### DLA-DRB1 genotyping

**Fig. 1:**
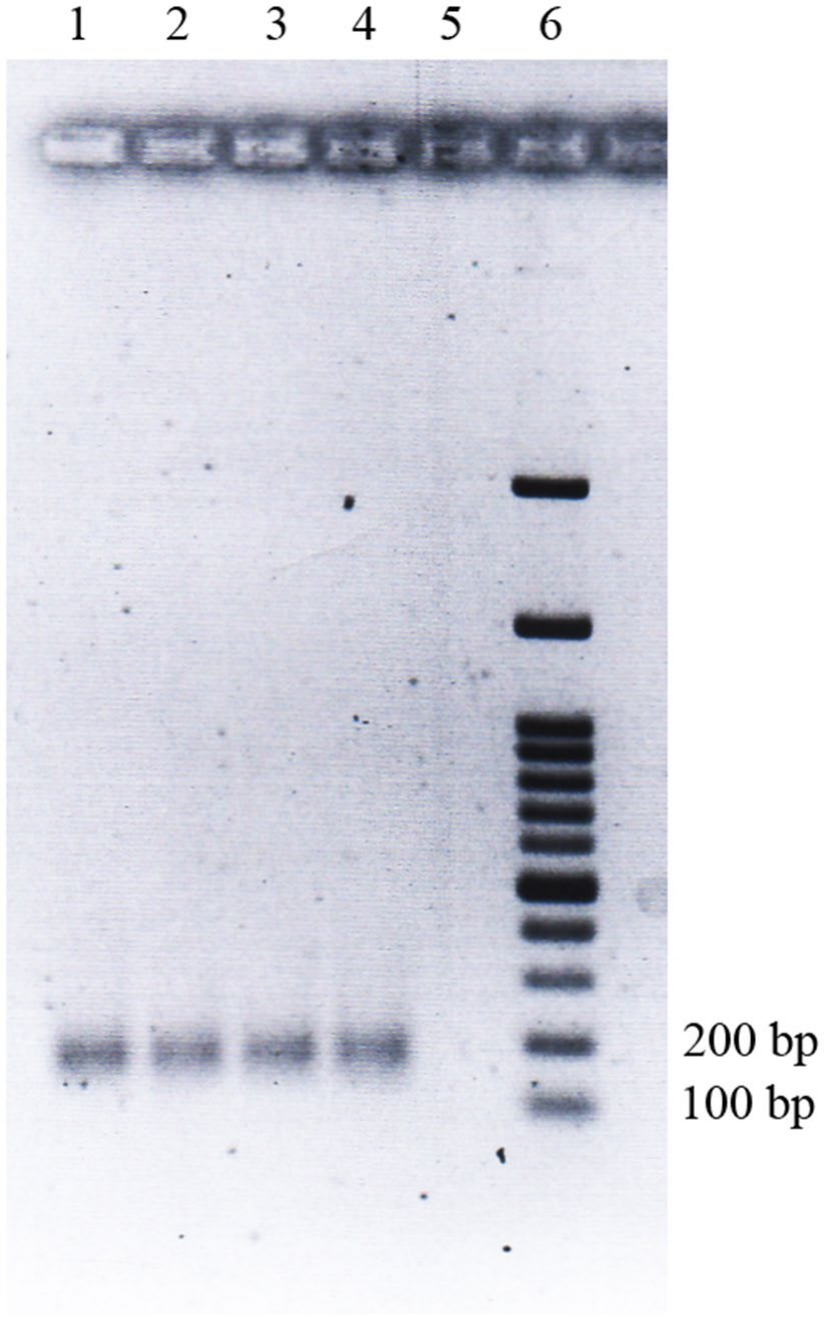
Polymerase chain reaction amplification of the DLA-DRB1.2 gene, separated by electrophoresis on 2% agarose gel. Lanes 1 and 2 show 138 bp fragment produced by CMT cases; lanes 3 and 4 contain 138 bp fragment produced by control group; lane 5 shows negative control; and lane 6 contains 100 bp DNA ladder

The second exon of DLA-DRB1 locus (DLA-DRB1.2) was amplified by nested polymerase chain reaction (PCR) and used for high resolution melting analysis (HRM) [21]. The first PCR step was performed in a final volume of 25 μL containing 20 ng genomic DNA, 1.5 mM Mgcl_2_, 200 μM of each dNTP, PCR buffer (20 mM Tris–HCl pH 8.4, 50 mM KCl), 1 U/μL of Taq DNA polymerase (CinaClon, Iran), and 20 pmol of DRBF (5′-GATCCCCCCGTCCCCACAG-3′) and DRBR3 (5′-CGCCCGCTGCGCTCA-3′) primers, resulted in the amplification of 303 bp DNA fragment. The thermal cycling profile for the first round of PCR was: 1 cycle of 95 °C for 2 min, 15 touch-down cycles of 95 °C for 30 s, followed by 30 s annealing, starting at 65.3 °C and reducing by 0.5 °C each cycle and 72 °C for 30 s. Subsequently, 1 µL of the first round PCR products was used as template DNA for the second round PCR in a final volume of 25 µL containing 20 pmol of DRBTF (5′-CCCCCGTCCCCACAG-3′) and DRBTR1 (5′-CCCCCACGTCGCTGTC-3′) primers, resulted in the amplification of 138 bp DNA fragment. For HRM analysis, 1 μL of EvaGreen^®^ Dye (2.5 mM) (Biotium, Darmstadt, Germany) was added to second step PCR master mix. The thermal cycling profile for the second round of PCR was: 1 cycle of 95 °C for 2 min, 30 cycles of 95 °C for 30 s, 59.8 °C for 30 s and 72 °C for 20 s, and a final extension of 72 °C for 5 min. 5 µL of the last PCR stage was electrophoresed on 2% agarose gels in order to check the quality and specificity of DNA fragment amplification (Fig. 1).

### High-resolution melting and melt curve analysis

HRM and melt curve analysis was performed in a Rotor-Gene™ 6000 thermal cycler (Corbett Life Science Pty Ltd, Sydney, Australia). In order to determine the optimal melting condition for differentiation of DLA-DRB1 alleles, the PCR products were setup on 0.2 °C/s ramping between 50 and 95 °C. The melting profiles were analyzed using Rotor-Gene 1.7 software and the HRM algorithm provided. Identical graphs classified in same groups with 90% confidence percentage. Homozygosity or heterozygosity was characterized by presence of one or two thermal picks and confirmed by the graphs of HRM.

### Sequencing

One sample from each HRM profile was sequenced to confirm the HRM results. Purification of PCR products and sequencing was performed by Macrogen Inc. (Seoul, Republic of Korea) on an ABI 3730 XL automatic DNA sequencer (Applied Biosystems, Canada). Sequences were aligned using BioEdit software v7.2.0 and compared with other DLA-DRB1 sequences from IPD database (http://www.ebi.ac.uk/ipd/mhc/dla/index.html).

### Statistical analysis

The effective allele numbers, genotype and allele frequencies, observed and expected homozygosity and heterozygosity for DLA-DRB1 locus were estimated using Popgene software [22]. The association between the DLA-DRB1 genotype and CMT profiles was determined as the odds ratio (OR). Frequency distribution of the genotype in each profile was compared using a general Chi squared test to examine the contribution of each genotype to the total Chi squared value. For genotype with a low or zero frequency, the denominator would be zero. In these cases, the OR would be undefined and Haldane`s modified Woolf formula was used for OR. The Fisher’s exact test was done to determine if the OR was statistically significant or not. An odds ratio value higher than 1.0 indicated that those dogs carrying the genotype were at lower risk of developing the CMT and were considered resistant to tumor. An odds ratio lower than 1.0 showed that those dogs carrying that genotype were at increased risk of developing CMT and were considered susceptible. Data were analyzed using the SPSS software version 21 and probability of P< 0.05 was considered statistically significant (SPSS Institute, Chicago, IL, USA).

## Results

### DLA-DRB1.2 genotyping

**Fig. 2:**
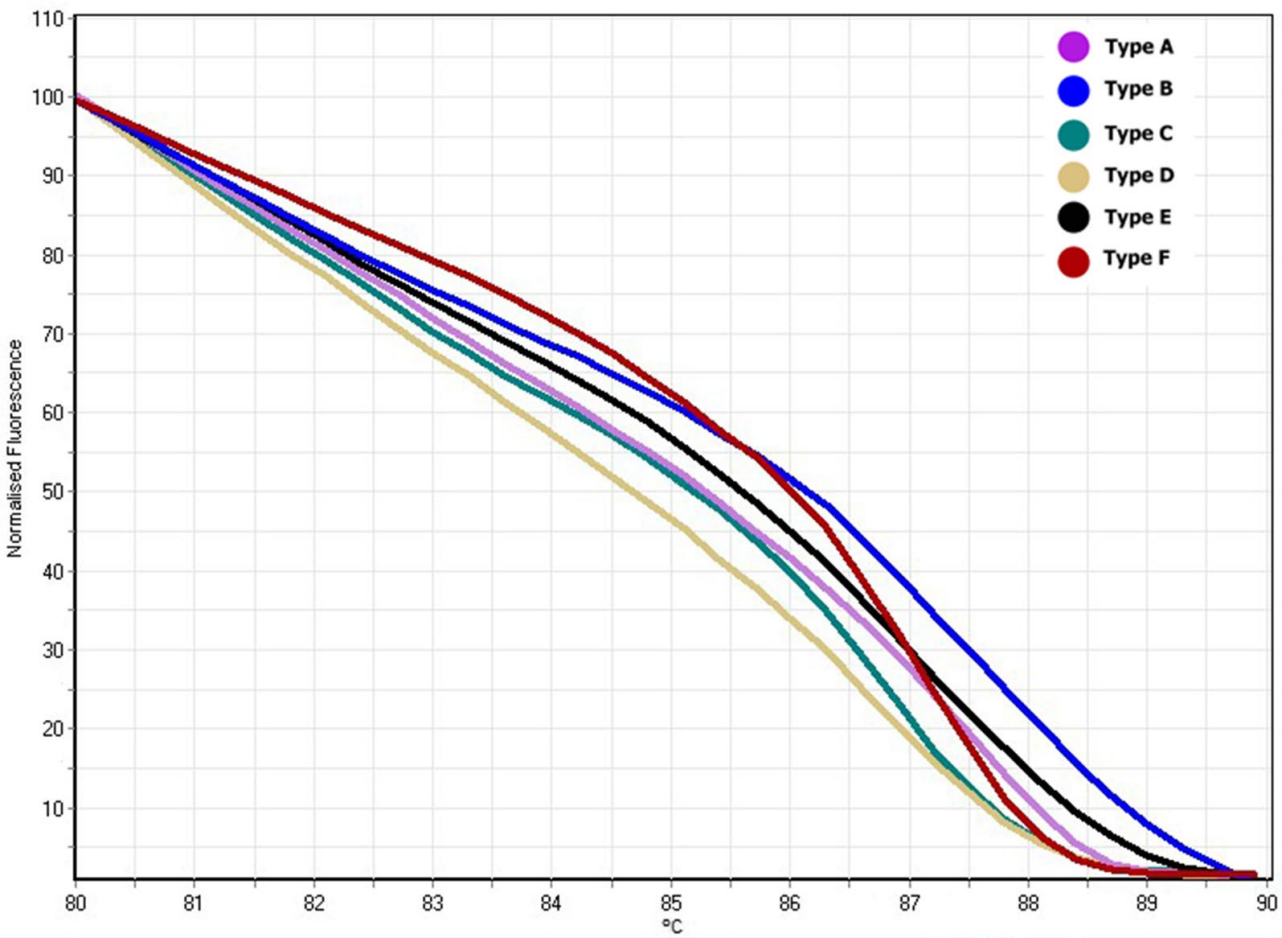
DLA-DRB1.2 HRM profiles and alignment of genotype sequences

**Fig. 3:**
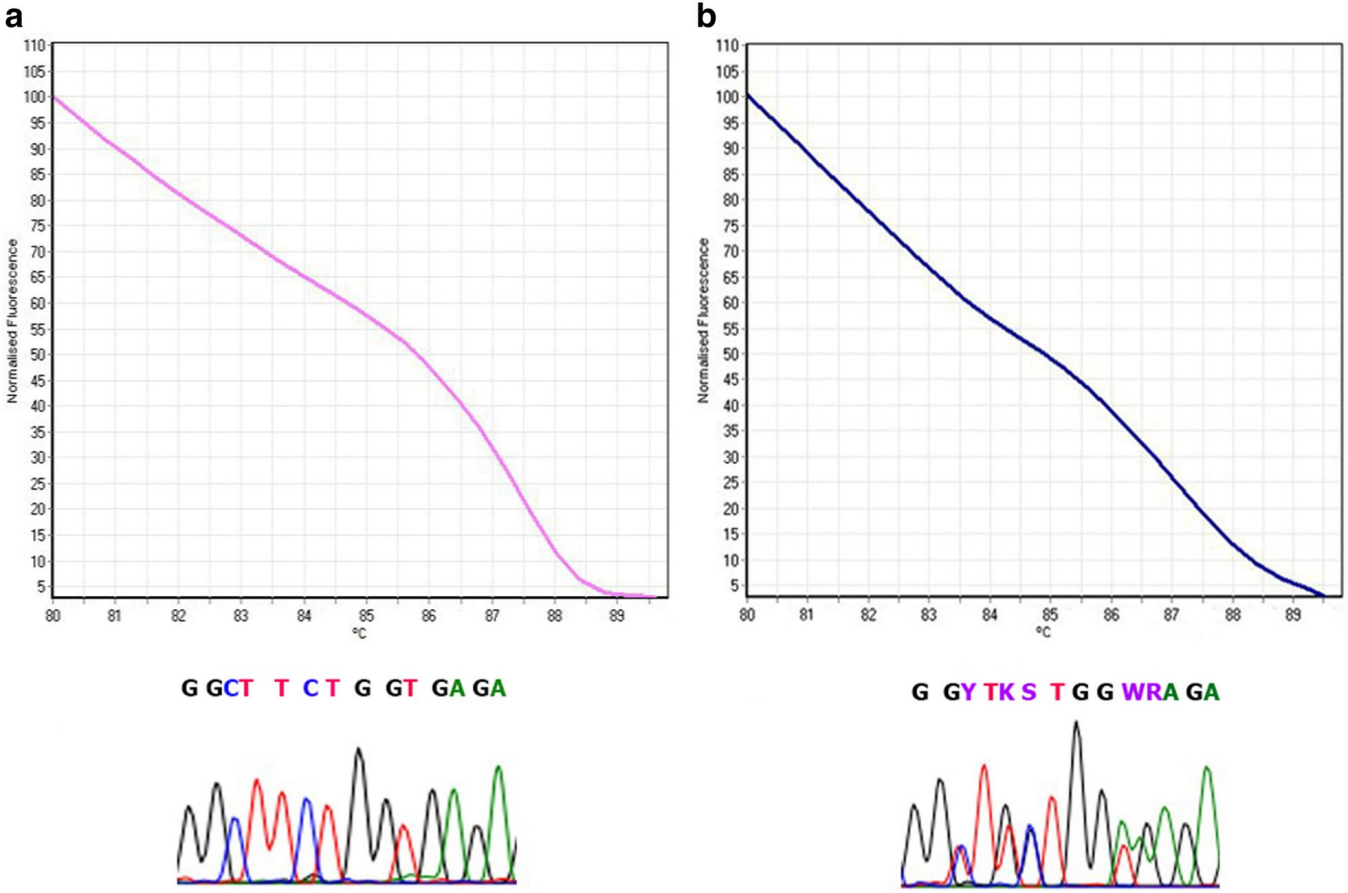
Confirmation of HRM profiles through sequencing in two homozygote (**a**) and heterozygote (**b**) samples

**Fig. 4:**
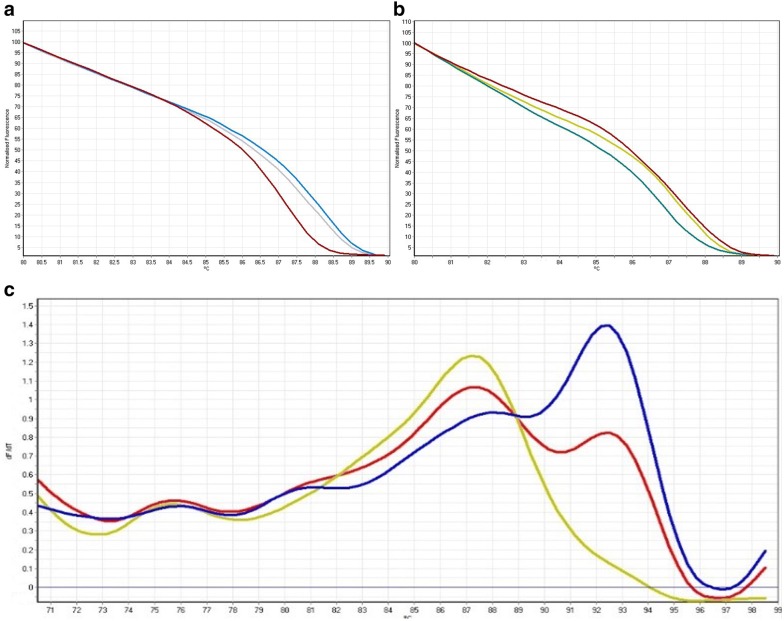
Homozygote (**a**) and heterozygote (**b**) HRM profiles. In the melt curve analysis (**c**), the blue and red lines represent heterozygotes and the yellow line represents a homozygote

HRM and melt curve analysis demonstrated eight patterns (A–H) in the 40 dogs (Fig. 2). The distribution of the DLA-DRB1.2 genotype frequencies related to each pattern is shown in Table 2. Genotype E showed the highest (22.2%) and genotype H the lowest (5.6%) frequency in the CMT group. In the control group, genotype A was the most (36.4%) and genotypes B and D the least (4.5%) frequent patterns, respectively (Table 2). Homozygote and heterozygote patterns identified through HRM analysis were confirmed by sequencing (Fig. 3). The observed heterozygosity (95.5) of DLA-DRB1.2 loci was significantly greater than the observed homozygosity (4.5%) in the control group (P ≤ 0.01). Results from the current study also revealed high level of heterozygosity in the tumor group, in which the proportion of heterozygous individuals was 66.7% (Fig. 4).

**Table 2:** DLA-DRB1 genotype frequencies and significant association of DLA genotypes and canine mammary tumor (CMT) between CMT cases and healthy controls

DLA-DRB1 genotype	CMT (n = 18)	Controls (n = 22)	Odds ratio (95% CI)	P-value
A	2 (11.1%)	8 (36.4%)	0.219	0.069
B	3 (16.7%)	1 (4.5%)	4.2	0.23
C	3 (16.7%)	3 (13.6%)	1.267	0.565
D	1 (5.6%)	1 (4.5%)	1.235	0.704
E	4 (22.2%)	2 (9.1%)	2.857	0.238
F	2 (11.1%)	3 (13.6%)	0.792	0.598
G	2 (11.1%)	2 (9.1%)	1.25	0.617
H	1 (5.6%)	2 (9.1%)	0.588	0.577

### Association of DLA-DRB1.2 genotypes with CMT profiles

No significant association was observed between the DLA-DRB1.2 genotypes and type of CMT (P > 0.05) (Table 2). However, the association study revealed a significant correlation between DLA-DRB1.2 genotypes with different CMT profiles. The E genotype was significantly associated with increased risk of having a carcinoma arising in a benign tumor with high significant relationship (P = 0.008; OR = 0.030) and B genotype represented a positive correlation with complex carcinoma (P = 0.013; OR = 0.04) (Table 3). Statistical analysis also revealed a significant association between the heterozygosity of DLA-DRB1.2 genotypes and presence of a CMT. DLA-DRB1.2 heterozygote genotypes were associated with lower risk of having a CMT (P = 0.024; OR = 10.526), while dogs with homozygote genotypes may develop mammary gland tumors more frequently. Association analysis between dog breed and CMT profiles indicated that the Terrier breed was more susceptible to development of a simple carcinoma mammary gland tumor (P = 0.042; OR = 0.789) than other breeds.

**Table 3:** Association of DLA-DRB1.2 genotypes with canine mammary tumor (CMT) profiles

CMT profiles	Fisher’s exact test (P-value)				Odds ratio (95% CI)			
	Complex carcinoma	Simple carcinoma	Carcinomas in benign tumor	Special types of carcinomas	Complex carcinoma	Simple carcinoma	Carcinomas in benign tumor	Special types of carcinomas
A	0.428	0.744	0.300	0.558	2.252	1.000	1.153	1.071
B	*0.013*	0.645	0.645	0.808	*0.041*	1.124	1.124	1.059
C	0.721	0.100	0.507	0.719	1.071	0.125	1.133	1.062
D	0.677	0.808	0.192	0.901	1.225	1.117	0.085	1.055
E	0.721	0.507	*0.008*	0.719	1.071	1.113	*0.030*	1.062
F	0.361	0.427	0.573	0.237	1.250	0.374	1.128	0.117
G	0.552	0.645	0.645	0.192	0.599	1.124	1.124	0.085
H	0.552	0.723	0.723	0.854	1.233	1.121	1.121	1.057

Italics font indicates statistically significant differences (P < 0.05)

## Discussion

Mammary gland tumors are the most common tumor in bitches with the incidence rate of about 25%. Among different factors involved in the development of CMTs, genetic background plays the leading role and can be considered as an expedient approach for early diagnosis of tumor and planning strategies towards having resistant populations [23]. However, selection based on genetics with a view of improving immunity and disease resistance needs powerful genetic markers linked to the immune system [24]. MHC class I molecules present endogenous antigens such as tumor peptides to cytotoxic T lymphocytes and mediate cellular immunity against tumor development [12, 25]. On the other side, class II molecules present exogenous antigens to helper T lymphocytes and induce humoral immune responses. Evidences suggest that MHC class II genes may play an important role in developing the risk of different tumors including cutaneous T cell lymphoma [26], renal cell carcinoma [11], breast cancer [19, 27], melanoma [13], acute leukemia [14], cervical cancer [15], and colorectal carcinoma [16] and any changes to the molecular structure of this class could be related to these neoplasms. Nevertheless, it is unclear that MHC II alleles in themselves have a causative effect on the development of tumors or whether they are the markers showing indirect association with the causative genes located in their vicinity and are in linkage disequilibrium with them.

MHC class II genes are appropriate candidates for studying the molecular genetic factors that may be associated with development of CMTs in different populations [12, 17–19, 27]. In the current study, we report the DLA-DRB1.2 polymorphism in a dog population and its association with mammary gland tumor profiles. DLA genetic diversity was evaluated using HRM technique and DNA sequencing method. In total, eight HRM patterns with a high level of heterozygosity were identified. Sequencing data also confirmed the HRM results regarding the homozygosity and heterozygosity of genotypes. HRM technique has been applied successfully for HLA genotyping in recent years [28–30]. Results of this study also propose that HRM technique could be considered as an appropriate MHC genotyping method in dogs, particularly when combined with DNA sequencing. HRM is able to identify homozygote or heterozygote genotypes in a fast, reliable and inexpensive way. However, more accurate and powerful approaches such as sequencing might be complementary to confirm the exact alleles.

The association study revealed a significant correlation between DLA-DRB1.2 genotypes E and B with susceptibility to the CMT profiles including carcinomas arising in a benign tumor and complex carcinomas. DLA class II loci have been associated with a number of canine autoimmune diseases including pancreatic acinar atrophy [31], hypoadrenocorticism (Addison’s disease) [32], CSK [9], necrotising meningoencephalitis [33], systemic lupus erythromatosus-related diseases [7], immune‐mediated hemolytic anemia [5] and anal furunculosis [34]. Association of DLA loci with tumor development has been estimated but to a limited extent. Aguirre‐Hernández et al. [35] studied the association between anal sac gland carcinoma with MHC class II loci (DLA-DRB1, DLA-DQA1, DLA-DQB1) in English Cocker Spaniels and showed significant correlations. The DLA-DQB1*00701 allele was associated with susceptibility for developing the carcinoma, while allele DLA-DQB1*02001 was resistant. DLA-DRB1 allele frequency showed no significant differences between cases and controls. In spite of the confirmed role of inflammatory responses in development of CMTs [36], there is no information regarding the MHC class II genotypes and CMT resistance. Therefore, the present study may highlight the significance of MHC molecules in developing carcinomas arising in a benign tumor and complex carcinomas.

The data revealed that DLA-DRB1.2 homozygote genotypes may have the higher risk of developing CMTs. These results are in agreement with a previous study, which reported that homozygosity in MHC class II genes increased the risk for developing CSK. Barrientos et al. [9] showed that dogs homozygous for the DLA-DRB1*69 allele had a risk for developing CSK that was over four times the risk of heterozygotes [9]. Dobermans with homozygous DLA-DRB1*00601, DLA-DQA1*00401 and DLA-DQB1*01303 genotypes were also more susceptible to Doberman hepatitis [37]. According to the heterozygosity advantage theory, homozygosity at MHC genes restricts the host’s ability to present a wider repertoire of immunogenic peptides to the immune system and leads to inefficient immune responses that increase the risk of tumor.

Considering the association between dog breeds and risk of having a CMT, no significant correlation was observed in this study. Salas et al. [38] revealed that Poodle, Cocker Spaniel, German Shepherd and Labrador Retriever were the most popular breeds affected by CMTs [38]. In another study conducted on occurrence and distribution of neoplasms in dogs, CMTs were reported in Poodle, Maltese, Chihuahua, Beagle, Yorkshire Terrier, Bichon Frisé, Cocker spaniel, English Springer Spaniel, Setter, Hound and German Shepherd breeds [39].

## Conclusions

The possible role of DLA-DRB1 gene polymorphism in developing CMT is reported. Statistically significant differences were observed in the distribution of DLA-DRB1 genotypes between different CMT profiles. The two most common genotypes in the tumor group (E and B) were associated with carcinomas arising in a benign tumor and complex carcinoma profiles, respectively. Findings of this study provide additional support for the association between MHC class II genes and development of CMT. Although this association can primarily explain the genetic basis of CMT, other susceptibility genes may also contribute to the development of these kind of tumors and need to be further examined. These results could potentially be used for early recognition of an increased risk of developing CMT and identifying susceptible dogs that require monitoring. However, it is worth noting that conclusions were made from a hypothesis generating study and more confirmatory studies needed.

## Data Availability

The datasets used and/or analysed during the current study are available from the corresponding author on reasonable request.
